# Targeting Endosomal Recycling Pathways by Bacterial and Viral Pathogens

**DOI:** 10.3389/fcell.2021.648024

**Published:** 2021-03-04

**Authors:** Xin Yong, Lejiao Mao, Xiaofei Shen, Zhen Zhang, Daniel D. Billadeau, Da Jia

**Affiliations:** ^1^Key Laboratory of Birth Defects and Related Diseases of Women and Children, Department of Paediatrics, West China Second University Hospital, State Key Laboratory of Biotherapy, Sichuan University, Chengdu, China; ^2^Hospital of Chengdu University of Traditional Chinese Medicine, Chengdu, China; ^3^Division of Oncology Research and Schulze Center for Novel Therapeutics, Mayo Clinic, Rochester, MN, United States

**Keywords:** endosomal recycling, pathogenic bacteria, human papillomavirus, SARS-CoV-2, retromer, WASH complex, TBC1D5, SNX

## Abstract

Endosomes are essential cellular stations where endocytic and secretory trafficking routes converge. Proteins transiting at endosomes can be degraded via lysosome, or recycled to the plasma membrane, trans-Golgi network (TGN), or other cellular destinations. Pathways regulating endosomal recycling are tightly regulated in order to preserve organelle identity, to maintain lipid homeostasis, and to support other essential cellular functions. Recent studies have revealed that both pathogenic bacteria and viruses subvert host endosomal recycling pathways for their survival and replication. Several host factors that are frequently targeted by pathogens are being identified, including retromer, TBC1D5, SNX-BARs, and the WASH complex. In this review, we will focus on the recent advances in understanding how intracellular bacteria, human papillomavirus (HPV), and severe acute respiratory syndrome coronavirus 2 (SARS-CoV-2) hijack host endosomal recycling pathways. This exciting work not only reveals distinct mechanisms employed by pathogens to manipulate host signaling pathways, but also deepens our understanding of the molecular intricacies regulating endosomal receptor trafficking.

## Introduction

Endocytosis is a key cellular step that mediates chemical information exchange between a cell and its extracellular environment. Integral membrane proteins, including receptors, ion channels, and solute carriers, are internalized during endocytosis either continuously or upon ligand binding (Grant and Donaldson, 2009). These proteins, along with their associated proteins and lipids (referred to as “cargo”), are subjected to distinct fates: some are targeted to lysosomes for degradation (degradation pathway), whereas others can be further delivered to the trans-Golgi network (TGN) or the plasma membrane for reuse (recycling pathway) (Burd and Cullen, 2014; Cullen and Steinberg, 2018; Wang et al., 2018). The choice of degradation or recycling pathways, known as endosomal sorting, not only determines the fate of cell surface proteins, but also impacts numerous signaling pathways. As a consequence, dysregulation of endosomal sorting has been implicated in a wide range of human diseases, such as cancer, neurological disorders, and diabetes (Small and Petsko, 2015; McMillan et al., 2017; Tu et al., 2020).

Recent work has discovered multiple adaptor and coat proteins that recognize specific amino acid sequences on the cytosolic tail of cargos destined for recycling, such as the retromer (Seaman et al., 1998), retriever (McNally et al., 2017), and multiple sorting nexin (SNX) proteins (Temkin et al., 2011; Steinberg et al., 2013; Simonetti et al., 2019; Yong et al., 2020). These proteins are spatiotemporally recruited to endosomes through sophisticated mechanisms involving the interaction with cargo, phospholipids, and small GTPases. For instance, retromer is targeted to endosomes through synergic actions of activated Rab7a, SNX3, and specific cargos (Harrison et al., 2014). Following the recruitment of coat proteins, cargos are subsequently packaged into tubulo-vesicular transport carriers, which then undergo scission form endosomes for targeting to the TGN or the plasma membrane. Many additional proteins, such as WASH and CCC (COMMD/CCDC22/CCDC93) complexes, TBC1D5, and VARP facilitate this process via distinct mechanisms, including tubule elongation, scission, and vesicle transport (Derivery et al., 2009; Gomez and Billadeau, 2009; Jia et al., 2010, 2016; Hesketh et al., 2014; Phillips-Krawczak et al., 2015).

Remarkably, recent studies have also uncovered that many intracellular bacteria, including *Chlamydia trachomatis* (Mirrashidi et al., 2015), *Legionella pneumophila* (Finsel et al., 2013), *Salmonella Typhimurium* (D’Costa et al., 2019), *Burkholderia cenocepacia* (Walpole et al., 2020), and *Coxiella burnetii* (Martinez et al., 2020), target host endosomal recycling pathways for their survival and replication (Table 1). These bacteria pathogens utilize different types of secretion systems and a plethora of effector proteins to create a unique vacuolar compartment known as pathogen vacuoles or inclusion, in which they can replicate. Similarly, some viruses, including human immunodeficiency virus (HIV) (Groppelli et al., 2014; Bayliss et al., 2020), hepatitis C virus (HCV) (Yin et al., 2016), human papillomavirus (HPV) (Lipovsky et al., 2013; Ganti et al., 2016), and severe acute respiratory syndrome coronavirus 2 (SARS-CoV-2; Cattin-Ortolá et al., 2020; Daniloski et al., 2020; Gordon et al., 2020), also interact with components of the endosomal recycling machinery during infection (Table 1). In this review, we will discuss recent advances in understanding how pathogenic bacteria subvert essential endosomal recycling pathways in the host. Subsequently, we use HPV and the emerging SARS-CoV-2 as examples to illustrate how viruses manipulate host endosomal recycling pathways to promote their infection, and highlight some common themes shared by bacterial and viral pathogens. Owing to the space limitations, we are only able to cover limited aspects of this exciting field. Interested readers are referred to other recent excellent reviews that delve further into these endosomal recycling pathways, processes, and impact by pathogenic factors (Personnic et al., 2016; Allgood and Neunuebel, 2018; Elwell and Engel, 2018; Siddiqa et al., 2018; Swart and Hilbi, 2020).

**TABLE 1:** Summary of pathogen effectors and host targets.

**Pathogen**	**Effector**	**Host target**	**Function**	**References**
**Bacteria**				
Chlamydia trachomatis	IncE	SNX5/6/32	Inhibits SNX-BAR activity	Mirrashidi et al., 2015; Elwell et al., 2017; Paul et al., 2017; Sun et al., 2017
	CT229	Multiple Rab GTPases	Interferes with trafficking of transferrin and CI-MPR	Rzomp et al., 2006; Faris et al., 2019
*Legionella pneumophila*	RidL	retromer	Inhibits retromer activity	Finsel et al., 2013; Barlocher et al., 2017; Romano-Moreno et al., 2017; Yao et al., 2018
*Burkholderia cenocepacia*	TecA	WASH	Delays phagosome maturation via F-actin	Walpole et al., 2020
*Salmonella Typhimurium*	SseC	retromer	Inhibits retromer activity	Sixt et al., 2017
	SifA	BLOC-2	Helps to position of Salmonella-containing vacuoles	D’Costa et al., 2019
	SifA	TBC1D5	Unknown	D’Costa et al., 2019
*Coxiella burnetii*	Cstk1	TBC1D5	Promotes replication of *C. burnetii*	Martinez et al., 2020
**Viruses**				
Human papillomaviruses (HPVs)	L2	Retromer	Promotes the intracellular transport of HPV	Lipovsky et al., 2013
	L2	SNX17	Promotes the entry of the virus	Bergant Marusic et al., 2012; Bergant and Banks, 2013
	L2	TBC1D5	Helps assembly and disassembly of retromer via promoting Rab7 cycling	Xie et al., 2020
SARS-CoV-2	NSP2	WASH, CCDC22	Likely interacts with endocytic pathway proteins to promote infection	Cattin-Ortolá et al., 2020; Daniloski et al., 2020; Gordon et al., 2020
	NSP7, NSP12, NSP16	Rab GTPase		
	Spike protein	SNX27		
Hepatitis C virus	NS5A	VPS35	Recruits retromer to promote HCV replication	Yin et al., 2016
*Herpes saimiri* virus	TIP	VPS35	Inhibits retromer activity	Kingston et al., 2011
Human immunodeficiency virus 1	Env	retromer	Delivers Env at the TGN	Groppelli et al., 2014

## *Chlamydia* Effector IncE and SNX-BARs

The SNX proteins are defined by the presence of a specific Phox (PX) homology domain, and are conserved from yeast to human (Teasdale and Collins, 2012; van Weering and Cullen, 2014). They are further classified into different subfamilies by the presence of additional domains, including BAR (Bin/amphiphysin/Rvs), FERM (protein 4.1/ezrin/radixin/moesin), and PDZ (postsynaptic density/disc large tumor suppressor/zona occludens) (Teasdale and Collins, 2012). One emerging concept in the field is that the PX domain can interact with a variety of proteins, in addition to phosphoinositides (Teasdale and Collins, 2012). This is best illustrated by the PX domain of SNX5/SNX6/SNX32, members of the SNX-BAR subfamily (Kvainickas et al., 2017; Simonetti et al., 2017, 2019; Yong et al., 2020).

**FIGURE 1:**
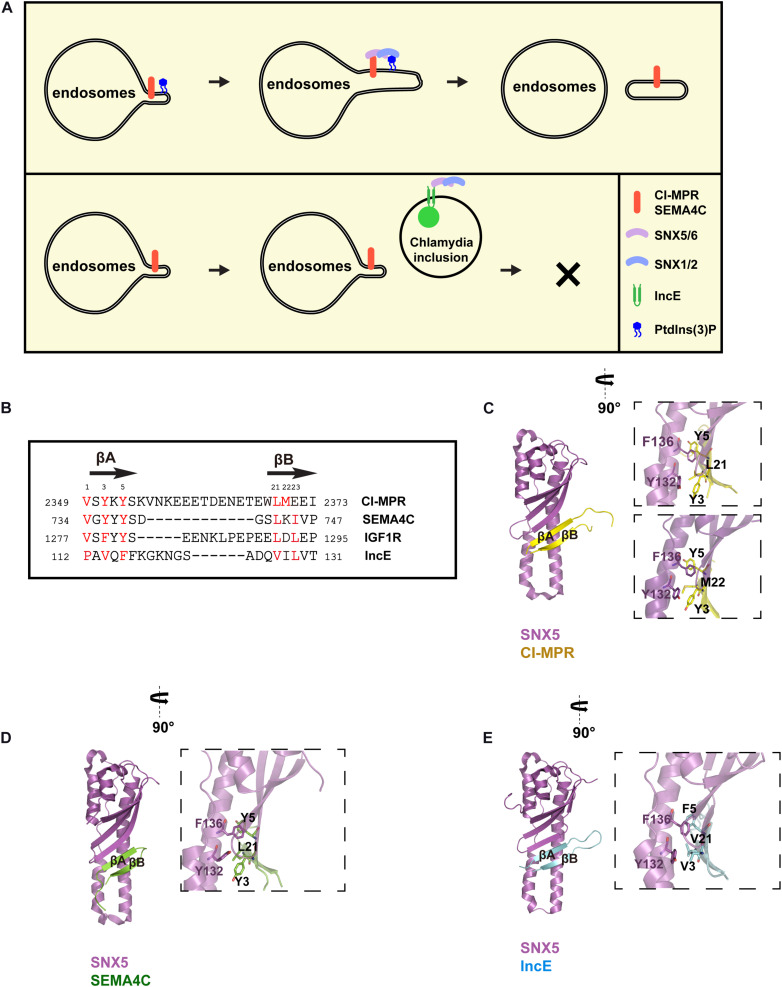
Recognition of SBM-containing cargos and IncE by SNX5. **(A)** SNX-BARs are recruited to endosomes through coincident detection of SBM by the PX domain of SNX5/SNX6, and PtdIns(3)P by the PX domain of SNX1/SNX2. SNX-BARs mediates endocytic recycling of SBM-containing cargos, such as CI-MPR and SEMA4C, which is disrupted by *Chlamydia* effector IncE. **(B)** Sequence alignment between IncE and several SBM-containing cargos, with secondary structure listed above. Numbers in two sides indicate the primary sequences of respective proteins, whereas numbers on the top denote the corresponding order of designated residues in this review. Red colors represent residues involved in binding to SNX5. **(C)** Interaction between the CI-MPR peptide (yellow) with the PX domain of SNX5 (violet). Two different crystal structures between CI-MPR and SNX5 have been determined, with L21 (top) or M22 (bottom) from CI-MPR inserting in the same hydrophobic pocket on SNX5. **(D)** Interaction between the SEMA4C peptide (green) with the PX domain of SNX5 (violet). **(E)** Interaction between IncE (cyan) with the PX domain of SNX5 (violet).

SNX5 or SNX6 (and likely SNX32) forms a stable heterodimer (referred as SNX-BARs herein) with SNX1 or SNX2 (Figure 1A). The PX domains of SNX1 and SNX2 preferentially recognize endosomal PtdIns(3)P and PtdIns(3,5)P_2_ lipids (Chandra et al., 2019). In contrast, the PX domains of SNX5 and SNX6 contain a unique insertion, and do not bind to PtdIns(3)P or PtdIns(3,5)P_2_ (Chandra et al., 2019). Recent studies from two independent groups have shown that SNX-BARs mediate recycling of cargoes like cation-independent mannose 6-phosphate receptor (CI-MPR) and Insulin-like growth factor 1 receptor (IGF1R), independent of retromer (Kvainickas et al., 2017; Simonetti et al., 2017). In the following studies, SNX-BARs and their cargos are found to directly interact with each other though the PX domain of SNX5/SNX6/SNX32 and a conserved bipartite motif, termed the SNX-BARs binding motif (SBM), within cargo proteins (Simonetti et al., 2019; Yong et al., 2020). Additional SBM-containing transmembrane proteins have been discovered to be recycled in an SNX-BAR-dependent manner, including SEMA4C and TRAILR1 (Simonetti et al., 2019; Yong et al., 2020). Dozens, if not hundreds, of additional SBM-containing proteins have been identified by proteomic or bioinformatics studies, although it remains to be experimentally determined whether they are indeed recognized and recycled by SNX-BARs (Simonetti et al., 2019; Yong et al., 2020). Finally, it should be noted that retromer (VPS35-VPS26A/B-VPS29 in higher eukaryotes) was originally shown to mediate endosome-to-TGN trafficking of CI-MPR (Arighi et al., 2004; Seaman, 2004, 2007). Possible reasons for this discrepancy and the role of retromer versus SNX-BARs in CI-MPR retrieval have been discussed elsewhere (Seaman, 2018; Cui et al., 2019), and thus will not be elaborated on here.

Intriguingly, SBM displays some degrees of sequence similarity with *Chlamydia* effector IncE (Figure 1B). *Chlamydia* is the leading cause of human respiratory, genital tract, and blinding eye infections. *Chlamydia* employs a large number of effector proteins to create the membrane-bound inclusion within host cells during infection (Moore and Ouellette, 2014). IncE is one of such effectors localized on the inclusion membrane. IncE directly binds to the PX domain of SNX5 or SNX6, but not that of SNX1 or SNX2 (Mirrashidi et al., 2015). This binding results in the re-distribution of SNX-BARs from endosomes to the inclusion membrane, blocking SNX-BARs-mediated CI-MPR trafficking (Mirrashidi et al., 2015; Elwell et al., 2017; Paul et al., 2017; Sun et al., 2017) (Figure 1A bottom). SNX-BARs were shown to restrict *Chlamydia* infection (Mirrashidi et al., 2015). Thus, IncE likely functions to block the inhibitory effect of SNX-BARs.

Crystal structures of the PX domain of SNX5 in complex with IncE or the SBM from CI-MPR or SEMA4C have been determined, providing the molecular basis how SNX-BARs recognize specific sequences (Elwell et al., 2017; Paul et al., 2017; Sun et al., 2017; Simonetti et al., 2019) (Figures 1C–E). Both SBM and IncE adopt a similar β-hairpin structure, and bind to the extended PX domain of SNX5. Residues from the upstream β-strand (βA) fit a strictly conserved Φx[F/Y/V]x[F/Y] consensus, where Φ and x represent aliphatic and any residues, respectively. Residues from the downstream β-strand (βB) fit a more loosely-conserved ΦxΦ motif. To facilitate discussion, the Φ residue from the Φx[F/Y/V]x[F/Y] consensus is designated as position 1, whereas the first Φ from ΦxΦ is positioned as 21 (Figure 1B). The hydrophobic residues from both strands of SBM or IncE, in particular residues at positions 3, 5, and 21, engage with a complementary hydrophobic groove of SNX5, predominately formed by residues Y132, L133, and F136 (Figures 1C–E). Interestingly, two different hydrophobic residues (L21 or M22) from CI-MPR pack against the hydrophobic pocket in two separate structures with CI-MPR, suggesting that a properly positioned hydrophobic residue from βB, rather than a specific residue, is critical for the binding (Figure 1C). Congruent with this structural information, incorporation of a single mutation within the WLM motif of CI-MPR did not dramatically impair its binding to SNX5; however, a triple alanine substitution (WLM/AAA) completely abolished the binding (Simonetti et al., 2019; Yong et al., 2020). The unique helices within the PX domain of SNX5 form part of the groove necessary for the binding, explaining the binding specificity of SNX5/SNX6/SNX32. Mutagenesis studies of SBM or SNX5 further confirmed the importance of cargo recognition for endosome-to-TGN or endosome-to-plasma membrane recycling (Simonetti et al., 2019; Yong et al., 2020). These studies nicely demonstrated that IncE inhibits SNX-BARs-mediated cargo trafficking through direct competition with cargoes.

IncE binds to the PX domain of SNX5/SNX6/SNX32 much more tightly than SBM-containing cargos in general. For instance, IncE and CI-MPR bind to the SNX5 PX domain with a dissociation constant (Kd) of 0.5–1 and 20 μM, respectively (Elwell et al., 2017; Paul et al., 2017; Sun et al., 2017; Simonetti et al., 2019; Yong et al., 2020). This difference could be partially explained by the length of the linker that connects two β-strands: the linker of CI-MPR is ∼5 residues longer than that of IncE (Figure 1B). When the linker of CI-MPR was shortened to ∼5 residues, its affinity toward to SNX5 increased sevenfold (Yong et al., 2020). Further shortening of the linker, however, decreased the binding. These experiments indicated that an optimal cargo of SNX-BARs likely has a linker of about 3–15 residues (Yong et al., 2020). This information will be helpful to identity additional SNX-BARs cargo proteins, and understand how endocytic recycling influences their cellular and physiological functions.

Apart from IncE, CT229 is another *C. trachomatis* effector that targets endosomal trafficking in host cells (Faris et al., 2019). Mechanistically, CT229 promotes the formation and maintenance of *Chlamydia* inclusion via the recruitment of multiple Rab GTPases and their effectors. Consequently, CT229 can modulate multiple host trafficking pathways, including trafficking of transferrin and CI-MPR (Rzomp et al., 2006; Faris et al., 2019). CT229 could also counteract the STING-mediated pro-death signal to prevent premature inclusion lysis and host death (Sixt et al., 2017). However, it is unclear whether such functions are related with its roles in endosomal trafficking.

## *Legionella* Effector RidL and TBC1D5/VARP

**FIGURE 2:**
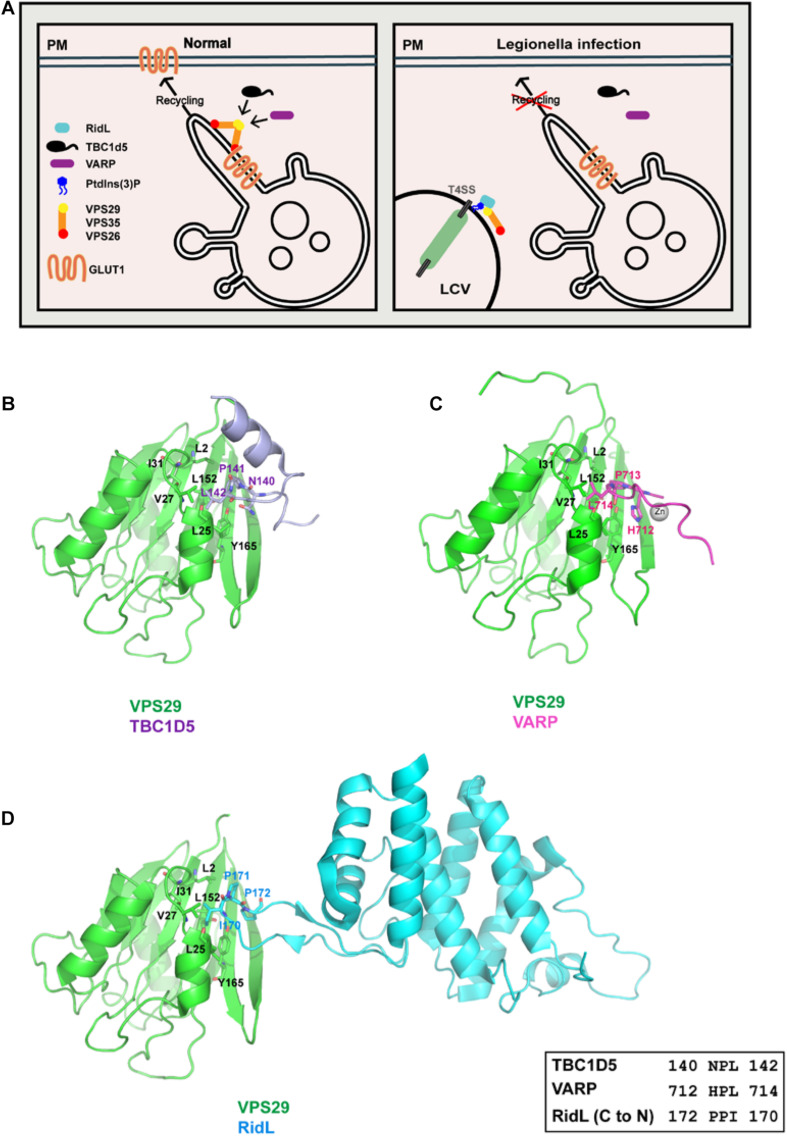
*Legionella* effector RidL directly binds the retromer subunit VPS29, outcompeting TBC1D5 and VARP binding. **(A)** Model depicting how RidL subverts retromer-dependent trafficking. Left: retromer mediates endosomes-to-plasma membrane trafficking, together with TBC1D5 and VARP. Right: *Legionella* employs type IV secretion systems (T4SSs) to translocate RidL and other effector proteins into host cells. RidL localizes on the LCV, probably via its ability to bind PtdIns(3)P. RidL directly binds to VPS29, and inhibits the binding of TBC1D5 or VARP to retromer. **(B)** Interaction between TBC1D5 (light blue) and VPS29 (green). **(C)** Interaction between VARP (hot pink) and VPS29 (green). **(D)** Interaction between RidL (cyan) and VPS29 (green). Inset shows alignment of VPS29-binding sequences from TBC1D5, VARP, and RidL.

Retromer is an evolutionarily conserved complex that can be found across nearly all eukaryotic organisms. Mammalian retromer consists of a VPS35-VPS26A/B-VPS29 heterotrimer and participates in both endosomes-to-TGN and endosomes-to-plasma membrane retrieval. Recent cryo-electron tomography studies of *Chaetomium thermophilum* retromer suggested that mammalian retromer might adopt a similar arch-shaped structure and extend away from membrane surface (Kovtun et al., 2018). Studies in the past have identified many regulators of retromer, including TBC1D5 and VARP (Figure 2A) (Seaman et al., 2009; Hesketh et al., 2014; McGough et al., 2014; Jia et al., 2016; Crawley-Snowdon et al., 2020). Interestingly, an effector from *L. pneumophila*, RidL, directly binds to retromer and competes with TBC1D5 and VARP (Figure 2A) (Finsel et al., 2013; Barlocher et al., 2017; Romano-Moreno et al., 2017; Yao et al., 2018; Crawley-Snowdon et al., 2020).

TBC1D5 belongs to the Tre2-Bub2-Cdc16 (TBC) family, and functions as GTPase-activating proteins (GAP) for both Rab7a and Rab7b (Mukhopadhyay et al., 2007; Seaman et al., 2009; Progida et al., 2010; Jia et al., 2016). As Rab7a is necessary for the endosomal targeting of retromer, TBC1D5 could function to inhibit the recruitment of retromer or promote the recycling of retromer (Seaman et al., 2009; Jia et al., 2016). TBC1D5 forms a tight complex with retromer, with a K_*d*_ of 220–450 nM (Jia et al., 2016). Two regions within the TBC domain of TBC1D5, termed Ins1 and Ins2, are necessary for retromer binding: Ins1 directly contacts VPS29, whereas Ins2 likely interacts with the N-terminus of VPS35 (Jia et al., 2016). Consistent with the tight binding, Steinberg and colleagues have shown that retromer and TBC1D5 cooperate to regulate lysosomal Rab7 activity and the mTOR signaling pathway (Jimenez-Orgaz et al., 2018; Kvainickas et al., 2019). In addition, TBC1D5 also has a role in regulating autophagy (Popovic et al., 2012; Popovic and Dikic, 2014).

VPS9-domain ankyrin repeat protein (VARP) is a multifunctional endosomal protein that interacts with membrane fusion regulator R-SNARE VAMP7, Rab32, Rab38, and retromer (Burgo et al., 2009; Hesketh et al., 2014; McGough et al., 2014). VARP is recruited to endosomes likely via its interaction with retromer component VPS29, Rab32-GTP, and Rab38-GTP (Hesketh et al., 2014; McGough et al., 2014). Functionally, Owen and colleagues showed that VARP mediates endosome-to-plasma membrane recycling of GLUT1, together with retromer (Hesketh et al., 2014). In contrast, Cullen et al. found that VARP is critical for the transport of a subset of retromer cargoes, including MCT1, CD97, and TRAILR1, but not GLUT1 (McGough et al., 2014). In short, although the exact functions of VARP in endosomal recycling are still under debate, VARP emerges as another critical regulator of this essential sorting pathway.

*Legionella pneumophila* is the prevalent causative agent of Legionnaires’ disease, a severe form of pneumonia. Once entering host cells, *Legionella* replicates in membrane-bound organelles, termed *Legionella*-containing vacuoles (LCVs). More than three hundreds of effectors are utilized to counteract host signal transduction and membrane dynamics to facilitate the infection (Newton et al., 2010). RidL is one such effector, which directly binds to the retromer subunit VPS29 as well as PtdIns(3)P (Finsel et al., 2013). Such an interaction promotes the accumulation of retromer on LCVs in infected cells (Finsel et al., 2013). As retromer inhibits *L. pneumophila* replication, RidL promotes bacterial replication via counteracting host retromer function.

Structural studies of VPS29 in complexed with RidL, or a small peptide fragment from TBC1D5 or from VARP have provided important insights into how VPS29 is recognized by diverse regulators and exploited by pathogens (Figures 2B–D) (Jia et al., 2016; Barlocher et al., 2017; Romano-Moreno et al., 2017; Yao et al., 2018; Crawley-Snowdon et al., 2020). All three proteins bind to a conserved hydrophobic pocket on the surface of VPS29, opposite to the VPS35 binding site. The binding involves in the “PL[I]” dipeptide from VPS29 ligands, and the hydrophobic pocket of VPS29 formed by L2, L25, V27, I31, L152, and Y165 (Figures 2B–D). The size of leucine/isoleucine side chain perfectly matches the size of the VPS29 hydrophobic pocket, explaining the requirement of leucine/isoleucine in the “PL[I]” dipeptide. The proline residue makes hydrophobic contacts with VPS29, and also helps to position leucine/isoleucine and other residues for optimal binding. Among all three proteins, RidL displays tightest binding toward VPS29 (K_*d*_ of 200–400 nM), consistent with its ability to compete with TBC1D5 or VARP (Barlocher et al., 2017; Romano-Moreno et al., 2017; Yao et al., 2018). Although TBC1D5 and VPS29 interact relatively weakly (Kd of ∼20 μM), TBC1D5 also binds to VPS35. As a result, TBC1D5 shows a much tighter binding to the retromer complex (Jia et al., 2016). VARP contains two CHPLCxCxxC sequences, with each of them binding to VPS29 with a K_*d*_ of 2–5 μM (Hesketh et al., 2014; Crawley-Snowdon et al., 2020). The two VPS29-binding motifs allow VARP to simultaneously bind to two adjacent VPS29 molecules in the assembled retromer coat (Crawley-Snowdon et al., 2020). Thus, VARP could also outcompete TBC1D5 despite its lower overall affinity to the VPS35–VPS26–VPS29 complex (Crawley-Snowdon et al., 2020).

Despite the similarities mentioned above, the three structures dramatically differ from each other in several ways. First, the three proteins show little primary sequence similarity outside of the “PL[I]” dipeptide. Second, the three VPS29 ligands adopt different secondary structures. Ins1 of TBC1D5 forms multiple sharp turns followed by a short α–helix, and most VPS29-interacting residues are from the turns (Figure 2B) (Jia et al., 2016). VARP possesses a unique “Zn-fingertail” formed by four cysteine residues and one Zn^2+^ (Figure 2C). RidL, on the other hand, utilizes a hairpin loop to contact VPS29 (Figure 2D). Lastly, despite the “PL[I]” dipeptide used in all three structures, the main chain direction of RidL is opposite to that of TBC1D5 and VARP (Figure 2D). Overall, these studies suggest that RidL outcompetes with TBC1D5 and VARP through structural mimicry. It remains to be determined whether other host or pathogenic proteins use a similar mechanism to interact with VPS29 and/or hijack the retromer activity.

## *B. Cenocepacia* Effector TecA and the WASH Complex

The pentameric WASH complex, consisting of WASH1, FAM21 (WASHC2), CCDC53 (WASHC3), Strumpellin and WASH1-interacting protein (SWIP or WASHC4), and Strumpellin (WASHC5), is a master regulator of endocytic recycling (Derivery et al., 2009; Gomez and Billadeau, 2009; Jia et al., 2010). Although it is missing in yeast Saccharomyces *cerevisiae*, it can be found in many other eukaryotic taxa (Wang et al., 2018). The complex predominantly localizes to endosomes, and promotes endosomal actin polymerization via activation of the Arp2/3 complex (Derivery et al., 2009; Gomez and Billadeau, 2009; Jia et al., 2010). WASH is required for efficient endocytic recycling of multiple cargo proteins from endosomes to TGN or to the plasma membrane, through cooperating with retromer or retriever (Derivery et al., 2009; Gomez and Billadeau, 2009; Puthenveedu et al., 2010; Zech et al., 2011; Gomez et al., 2012; Piotrowski et al., 2013; Phillips-Krawczak et al., 2015). Furthermore, recent studies showed that the WASH complex subunit FAM21 directly interacted with TGN-localized TBC1D23, thus mediating vesicle capturing at the TGN during endosome-to-TGN retrieval (Newton et al., 2010; Shin et al., 2017; Huang et al., 2019; Liu et al., 2020; Tu et al., 2020).

A recent study suggested that WASH activity was hijacked for phagosome maturation during *B. cenocepacia* infection (Walpole et al., 2020). *B. cenocepacia* is a major causative pathogen for serious pulmonary infections in immunocomprised patients. Early studies showed that *B. cenocepacia* formed a pathogen vacuole, which could mature to early phagosome but be resistant to fusing with lysosomes (Lamothe et al., 2007; Huynh et al., 2010). Its effector TecA functions to inactivate Rho GTPases RhoA and Rac1 through deamidation (Aubert et al., 2016). Recently, Walpole et al. (2020) further extended this study by showing that TecA also induced formation of filamentous actin (F-actin) around the phagosome, and host WASH protein was required for the formation of F-actin. Accumulation of F-actin delayed phagosome maturation, indicating that pathogenic bacteria could subvert WASH activity for their survival and replication. The mechanism by which RhoA and Rac1 inactivation via TecA leads to activation of WASH activity remains completely unclear and warrants further investigation.

## Manipulation of Host Endosomal Trafficking by *S. Typhimurium*

*Salmonella Typhimurium* is the major cause of food poisoning in Western countries (Mead et al., 1999). In order to establish infection, *S. Typhimurium* utilizes over 40 effectors to manipulate host cell signaling pathways (Figueira and Holden, 2012). To uncover new functions of bacterial effector proteins, Patrick et al. developed a quantitative genetic interaction profile methodology, termed E-MAP (Sixt et al., 2017). They found that *S. Typhimurium* protein SseC maintained the Salmonella vacuole through interactions with retromer components VPS35 and VPS26 (Sixt et al., 2017). Depletion of host retromer promotes *S. Typhimurium* replication, analogous to studies of *L. pneumophila*.

The above genetic approach is complemented by a proteomic study identifying effector–host proteins interaction. A recent study utilizing proximity-dependent biotin identification (BioID) has identified a large number of new host proteins that interact with some well-known *S. Typhimurium* effectors (D’Costa et al., 2019). For instance, BioID uncovered BLOC-2 and TBC1D5 as binding partners of SifA, in addition to its known binding partner SKIP (SifA- and kinesin-interacting protein), a host protein critical for vacuolar membrane dynamics (Beuzon et al., 2000; Ohlson et al., 2008; McEwan et al., 2015). The BLOC-2 complex, comprised of three proteins HPS3, HPS5, and HPS6, is critical for the biogenesis of lysosome-related organelles and melanosome maturation (Di Pietro et al., 2004). The SifA–BLOC-2 interaction is necessary for the positioning of Salmonella-containing vacuoles (D’Costa et al., 2019). Although ectopically produced SifA localizes on late endosomes and lysosomes, similar to TBC1D5, in particularly when co-transfected with another effector SseJ, the functional relevance of the SifA-TBC1D5 interaction remains to be established (Ohlson et al., 2008; D’Costa et al., 2019). Finally, multiple SNXs, including SNX1 and SNX3, also play critical roles during maturation of the Salmonella vacuole (Bujny et al., 2008; Braun et al., 2010). Thus, Salmonella hijacks multiple pivotal components of endocytic recycling pathways to establish its pathogen vacuoles.

## *Coxiella Burnetii* Effector CstK1 and TBC1D5

*Coxiella burnetii* is a highly infectious intracellular bacterium that causes the zoonosis Q fever, which has flu-like symptoms. An earlier genome-wide screen has demonstrated that multiple components of endocytic recycling pathways are required for *C. burnetii* growth (McDonough et al., 2013). Notably, retromer and several associated SNXs (SNX2, SNX3, SNX5, and SNX6) appeared to promote intracellular replication of *C. burnetii* (McDonough et al., 2013). These findings were in sharp contrast to results obtained from *L. pneumophila* and many other vacuolar bacteria, where the endocytic recycling machinery restricted bacterial growth (Finsel et al., 2013; Mirrashidi et al., 2015). The underlying mechanisms for this difference remain unclear.

More recently, the *C. burnetii* effector Cstk1 was shown to regulate the biogenesis of the pathogen vacuole (Martinez et al., 2020). Cstk1 is a Ser/Thr/Tyr kinase and interacts with TBC1D5. Furthermore, suppression of TBC1D5 inhibits the proliferation of *C. burnetii*. Surprisingly, CstK1 did not phosphorylate TBC1D5 *in vitro*. This interesting study raises several more interesting questions: (1) Does Cstk1 affect the GAP activity of TBC1D5? (2) Does TBC1D5 regulate the kinase activity of CstK? and (3) How does TBC1D5 impact the proliferation of *C. burnetii*, via regulating lysosomal activity, cargo trafficking, or autophagy?

## HPV L2 Protein Hijacks Both Retromer and SNX17 for Cellular Trafficking

**FIGURE 3:**
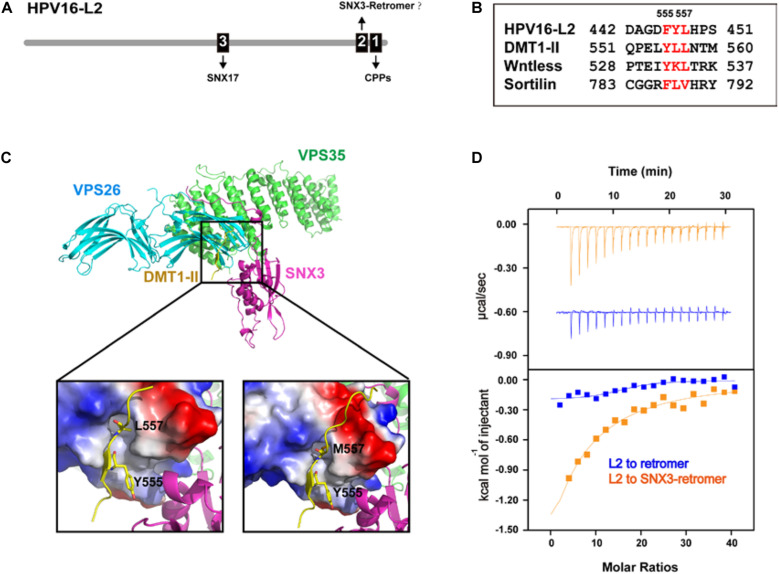
Cellular trafficking of HPV16 L2 may involve SNX3, in addition to retromer and SNX17. **(A)** A schematic diagram of HPV16-L2 showing CPP, SNX3–retromer, and SNX17-binding sites. **(B)** Alignment of HPV16 L2 retromer binding sequence and several known SNX3–retromer cargoes. **(C)** Crystal structure of the SNX3–retromer–DMT1-II complex shows that both VPS26 and SNX3 are involved in the recognition of the DMT1-II recycling motif. **(D)** ITC experiments of the L2 peptide titrated into retromer in the absence or presence of SNX3 in a buffer containing 100 mM Hepes pH 7.5 and 200 mM NaCl at 25°C. Top and bottom panels show raw and integrated heat from injections, respectively. The curves in the bottom panel represent a fit of the integrated data to a single-site binding model.

Human papillomaviruses cause about 5% of human cancer, in particular cervical cancer and oropharyngeal cancer (Wang et al., 2020). Over 200 types of HPV have been identified. Among them, the HPV16 and HPV18 subtypes are the most common high-risk strains (Royle et al., 2015; Xu et al., 2019). Although over 100 countries have introduced HPV vaccination programs (Drolet et al., 2019), no drugs are available that specifically inhibit HPV infection. HPVs are non-enveloped DNA viruses that contain both L1 major capsid protein and L2 capsid protein. The L2 protein plays an indispensable role for proper trafficking of the virus to the nucleus, where viral DNA replicates (Kamper et al., 2006; Buck et al., 2008; Campos, 2017). The L2 protein harbors a short sequence enriched of basic amino acids near its C terminus, known as a cell-penetrating peptide (CPP) (Figure 3A) (Guidotti et al., 2017). After HPV enters host cells, the CPP sequence guides a portion of L2 through the endosomal membrane into the cytoplasm. Adjacent to the CPP, L2 encompasses a sequence that directly binds to retromer (Figure 3A) (Lipovsky et al., 2013, 2015; Popa et al., 2015; Zhang et al., 2018). Although L2 is not a transmembrane protein, the binding of L2 by retromer helps the trafficking of the virus to the TGN en route to the nucleus. Mutation of CPP or retromer-binding site with L2 or depletion of retromer in cells delays exit of HPV from endosomes, thus inhibiting HPV infection (Zhang et al., 2018, 2020).

Interestingly, the retromer-binding sequence of L2 shares some similarity with those of several cargo proteins that are recognized by retromer and SNX3, including DMT1-II, Wntless, and Sortilin (Figure 3B) (Lucas et al., 2016; Zhang et al., 2018). Crystal structure of the VPS26–VPS35–SNX3–DMT1-II complex shows that the Φx(L/M) motif of DMT1-II adopts an extended conformation and is recognized by both VPS26 and SNX3 (Figure 3C) (Lucas et al., 2016). Interaction with SNX3 and DMT1-II induces a conformational change in VPS26, exposing the binding site for DMT1-II. L557 from DMT1-II (or its substitution M557) is completely buried within a hydrophobic pocket between strands β10 and β18 of VPS26 (Lucas et al., 2016). And the side chain of Y555 is embraced by multiple hydrophobic residues at the VPS26–SNX3 interface. Mutation of either L557 or Y555 decreases the recruitment of retromer to membranes, and impairs sorting of DMT1-II cargo out of endosomes (Lucas et al., 2016).

To test whether SNX3 may cooperate with retromer in binding L2, we performed isothermal titration calorimetry (ITC) to assess the interaction between HPV16-L2 and retromer in the absence or the presence of SNX3 (Figure 3D). The interaction between L2 and retromer was barely detected in the absence of SNX3. The addition of SNX3 greatly enhanced the interaction and resulted in a K_*d*_∼140 μM, similar to the binding affinity between DMT1-II and the SNX3–retromer complex (K_*d*_∼127 μM) (Lucas et al., 2016). It remains to be established that SNX3 is indeed involved in the intracellular trafficking of HPV.

In addition to retromer and possibly SNX3, SNX17 also participates in the endocytic trafficking of HPV16 L2 (Bergant Marusic et al., 2012). HPV16 L2 contains a _254_NPAY_257_ sequence N-terminal to the retromer-binding site, similar to the NPxY motif recognized by SNX17 (Figure 3A) (Bergant and Banks, 2013). Indeed, HPV16 L2 was shown to directly interact with SNX17. Interestingly, SNX17 does not affect viral entry or retention in early endosomes, but mediates the localization of HPV virions in late endosomes, suggesting that SNX17 may participate in the late stage of HPV16 entry. A different study has suggested another SNX17-binding site in HPV16 L2, _250_NPVY_254_. However, its functional relevance remains to be established. Finally, Pim et al. (2015) identified a PDZ-binding motif (PDZbm) in the central portion of HPV16 L2. Since the PDZ domain recognizes the C-terminus of a protein, this motif is unlikely to bind the PDZ domain of SNX27. Consistent with this notion, a recent study suggested that the interaction between HPV16 L2 and SNX27 is indirect, and may be mediated by other proteins (Pim et al., 2020).

## HPV L2 and TBC1D5

It has been known for some time that the cellular trafficking of HPV requires Rab7a (Day et al., 2013; Lipovsky et al., 2013; Young et al., 2019). How Rab7a mediates HPV trafficking remained unclear until recently. Using a protein interference screen, Xie et al. (2020) identified that TBC1D5 was essential to HPV entry. The recruitment of TBC1D5 to the retromer–L2 protein complexes likely leads to the disassembly of the retromer–HPV complex through inactivation of Rab7a-GTP, thereby promoting the recycling of retromer. Notably, Xie et al. demonstrated that the cycling of Rab7a-GTP and Rab7a-GDP is required for HPV trafficking. In contrast, the cellular trafficking of CI-MPR and DMT1-II requires only Rab7-GTP (Xie et al., 2020). These findings greatly expand our understanding the mechanisms by which HPV manipulates host cellular activity, and suggest that TBC1D5 inhibitors could suppress HPV infection.

## Summary and Outlooks

In the last several years, we have witnessed significant advances in understanding of the regulation of endocytic recycling. Many new factors have been identified, and novel functions have been uncovered for some classic endocytic recycling proteins (such as cargo recognition by SNX5/SNX6). Furthermore, it is increasingly clear that the molecular machines that facilitate and regulate endocytic recycling represent targets for pathogenic factors produced by both intracellular bacteria and viruses. Retromer, TBC1D5, some SNX proteins, and the WASH complex are among the endocytic recycling proteins that are frequently targeted by both bacterial and viral pathogens. Why do these pathogens target endocytic recycling pathways? One reason is that internalized viruses are transported from endosomes to downstream cellular compartments, following the same route of endocytosed proteins. Similarly, bacterial pathogens utilize components of endocytic recycling pathways to decorate their vacuoles, likely to mimic recycling endosomes in order to decrease their fusion with lysosomes or prevent lysosomal maturation.

Dissecting the mechanisms that pathogens use to target host factors not only teaches us the “tricks” used by pathogens to manipulate host pathways, but also helps to uncover some “hidden” properties of host proteins. For instance, long before SNX-BARs were shown as to directly interact with CI-MPR, IGF1R, TRAILR1, and other cargos to mediate their trafficking, the *Chlamydia* effector IncE was known to bind SNX5 and SNX6 (Mirrashidi et al., 2015). This observation along with structural information and binding studies revealed that the SNX5/SNX6 PX domains were not involved in phosphoinositide binding, but were involved in cargo recognition. With additional bacterial effectors and viral proteins being characterized, new mechanisms will likely be uncovered, including additional host regulators of endocytic recycling pathways or novel functions.

As an enveloped positive-sense RNA virus, SARS-CoV-2 has infected over 80 million people and caused the deaths of over 1.7 million people worldwide, as of December 2020. SARS-CoV-2 requires many host factors for all stages of its life cycle. Interestingly, multiple lines of evidence suggest that SARS-CoV-2 may also manipulate the endocytic recycling pathways in favor of its infection. First, a genome-scale CRISPR screen has identified multiple components in the endocytic recycling pathways required for SARS CoV-2 viral infection, including Rab7a, retromer, SNX27, and multiple members of CCC (Daniloski et al., 2020). Second, several proteomic studies have shown that multiple SARS CoV-2 proteins interact with host proteins that regulate endocytic recycling, including NSP2 and the WASH complex, NSP7, NSP12, NSP16, and CCDC22, and NSP7 and Rab protein (Gordon et al., 2020). Last, the cytoplasmic tail of SARS-CoV-2 spike protein can directly interact with SNX27 (Cattin-Ortolá et al., 2020). Although it is too early to say how SARS-CoV-2 interacts with endocytic trafficking proteins and interferes with their functions, it is very likely that SARS-CoV-2 engages the endocytic trafficking machinery to establish infection. Targeting of these critical endosomal receptor recycling machineries also likely alters the levels of ACE2, which is required for viral binding and cell entry.

Further research will be necessary to elucidate the molecular strategies that SARS-CoV-2 employs to facilitate its infection via manipulating endocytic recycling pathways. More importantly, our detailed understanding of the molecular mechanisms by which these bacterial and viral pathogenic factors affect endocytic recycling will likely result in the development of novel targeted therapeutics to prevent or resolve infections. In this regard, it is worthy to note some pioneering work by Stechmann et al. (2010) that have identified small molecules that showed efficacy against ricin in mice by selectively blocking endosome-to-Golgi trafficking of ricin toxin.

## Author Contributions

All authors prepared the manuscript. XY and ZZ performed ITC experiments in Figure 3D.

## Conflict of Interest

The authors declare that the research was conducted in the absence of any commercial or financial relationships that could be construed as a potential conflict of interest.
